# Supplementary material for: A sero-epidemiological analysis of Coxiella burnetii infection and its risk factors in livestock from Addis Ababa, Adama, and Modjo abattoirs and pastoral areas of Oromia, Ethiopia
Source: PLoS Negl Trop Dis. 2024 Jul 16;18(7):e0012287. doi: 10.1371/journal.pntd.0012287 (PMC11251603; doi:10.1371/journal.pntd.0012287)
Supplement: Attachment — Submitted filename: renamed_d9c72.docx [file pntd.0012287.s001.docx]

**Response to Reviewer’s comments**

First, we would like to thank the editor and reviewers for sparing their time in carefully going through the manuscript and providing scholarly comments that are believed to enrich the manuscript. Below are our point-by-point responses to the reviewer’s comments that are incorporated in the main manuscript.

**Reviewer #1:**

Comment #1: Regarding the manuscript entitled “A comprehensive analysis of Coxiella burnetii infection (Q fever) and its risk factors in livestock at Addis Ababa, Adama, and Modjo abattoirs, and Pastoral areas of Oromia, Ethiopia: a seroepidemiological study”, the authors aimed to estimate the seroprevalence of C. burnetii infection and associated risk factors in cattle, sheep, goats, and camels, respectively from Addis Ababa, Adama, and Modjo abattoirs and pastoral areas of Oromia, Ethiopia.

**Response:** Now the title is changed as per the recommendation

Comment #2: English editing is needed for the whole manuscript.

**Response**: The manuscript is edited as much as possible

Comment #3: The study used a preliminary method for the diagnosis of Q fever. There is no molecular investigation.

Samples were collected from abattoirs and three selected pastoral areas. Therefore, the authors may not have a trustworthy history of these animals. Were these animals suffering from a history of disease, abortion, fever,………………..?

**Response**: The animals sampled at the abattoirs were not suffering from illness but there is history of abortion and febrile illness in the herd/flock from which the animals originated. Establishment of differential diagnosis of abortion will be the next assignment

Comment #4: “All animals sampled were clinically healthy.” Did you examine all these animals clinically before slaughter? Did you confirm that their carcasses were accepted after visceral evaluation?

**Response**: yes the animals were clinically examined before slaughter. However, the examination of the carcass was done by the authorized meat inspection veterinarians. The results of the inspection showed that the carcasses were acceptable.

Comment #5: Why the author did not analyze the data in Table 1 statistically using chi-square?

**Response:** we did not do it because we made our final inference on the results of multivariable logistic regression where OR is used as measure of association. If it is necessary we can do it.

Comment #6: The authors must clarify the importance of these data for the international community.

**Response**: Now it is clarified

**Reviewer #2:**

This study is to investigate a seroepidemiology and risk factors of C. burnetii infection in four different species of animals in Ethiopia. This paper deserves to be published in Plos Journal. However, there are some critical concerns to make the reviewer hesitate to accept your article.

Major comments

Comment #1: - Q fever mean C. burnetii infection in human, whereas coxiellosis is animal disease caused by C. burnetii. This study was only done in animals, so Q fever should be removed from study title. This study is only a cross-sectional seroprevalence study even though it was tough works in that a large-scale of serum sample was collected from four different areas and animals. The study title is better to be “A seroepidemiological analysis of coxiella burnetii infection and its risk factors among livestock in Ethiopia: A nationwide study

**Response:** The title is modified as recommended

Comment #2: - Journal guideline doesn’t have word count limitation, but it is recommended to present the study findings CONCISELY. However, this paper has about the number of 6,900 word, and the number of references is 74. Needless repetitions and redundant descriptions are seen all over the manuscript. If the authors will be able to concise the original draft and omit needless repetitions and redundant descriptions, this manuscript seems to be a well-written article. For this, it would be better to pay attention at least my following comments. To present study finding concisely and study suggestion clearly, all authors should review original draft carefully, and try to clarify study background, results and discussion.

**Response:** Thank you very much. We tried to make it concise

Comment #3: - In introduction, the description for study background is too long. Three or four paragraphs will be enough to construct three parts in introduction. It would be better to concise or reduce it up to two third in current amount of introduction. This manuscript is not a review article, but a research article. The number of references in the introduction section is too many. The author should cite only necessary references up to about 15.

**Response:** Thank you a lot again. It is done now as per the recommendations given

Comment #4: - In methods, description of study area is only supplementary findings, not directly involved in study methods. It would be better to move into supplementary material called supporting information including figure 1.

**Response**: now it is moved

Comment #5: - In line 332-337 of result section, this paragraph is not the study finding but methodologic description. It is clearly an example of needless repetitions.

**Response**: It is removed now

Comment #6: - In study findings, one of some critical concerns is about “animals infested with ticks” How did the authors define “animals infested with ticks”? In this study, Tick infestation is the greatest risk factor of seropositivity in animals. It can make readers misunderstand that C. burnetii infection in animals seems to be tick-borne disease. I can agree that tick can contribute to transmission among livestock and wildlife. However, given that human Q fever is primarily transmitted from livestock and transmission through tick is VERY unusual, this finding is likely to be biased. It should clarify the definition of tick infestation and consider re-analysis on the basis of clear study definition.

**Response**: The comparison was between animals heavily infested (carrying ticks on several body parts) and those animals which completely free of ticks. This correction is made.

Comment #7: - In the section subtitled “Analysis of risk factors of C. burnetii infection”, table 2 seems to be univariate analysis on association between seropositivity of C. burnetii infection and relevant variables. The authors should discuss statistical analysis with a statistician, and address statistician opinions in the revision letter. I recommend that OR of multivariate analysis along with OR of univariate analysis should be shown in table.

**Response:** The results of analysis presented in Table 2 are not univariable analysis. It is multivariable analysis. The analysis is done by statistician. The OR presented is adjusted OR.

Comment #8: - In line 383-384, there are two different but similar subheading. Please avoid the redundant description.

- In line 383-413 subtitled “seroprevalence of C. burnetii at abattoris”, I think this section is not directly involved in study results, and it is much closer to background description of study area in the method section. Thus, it can move to supporting information. If the authors would like to remain these paragraphs in the result section, it should be removed the paragraphs of line 385-394 and line 409-413. Result section is for study findings, but not for methods and discussion.

**Response**: It is moved to the supplementary section as recommended.

Comment #9: - In the discussion section, first paragraph of line 439-445 offer little that is associated with study results. This paragraph just addresses universal characteristics of C. burnetii infection. It can be removed from discussion section, and second paragraph of line 446-451 is much better to be first paragraph of discussion section.

**Response:** Thank you very much for this. It is corrected now

Comment #10: - The sections of discussion and conclusion consist of 8 pages, and it is too long description.  Needless repetitions and redundant descriptions are also seen in the discussion. Thus, it would be better to concise or reduce it within 5 pages. Total number of references in this manuscript is 74, and it is too many. The author should cite only necessary references within 40.

**Response:** Now it is corrected

Comment #11: - The paragraph for recommendation should be removed from main manuscript. Previously said, the author should aware that this paper is only for research article, but not for narrative review.

**Response:** Thank you very much. Sorry for this silly mistake. It is corrected accordingly
