# Supplementary material for: A sero-epidemiological analysis of Coxiella burnetii infection and its risk factors in livestock from Addis Ababa, Adama, and Modjo abattoirs and pastoral areas of Oromia, Ethiopia
Source: PLoS Negl Trop Dis. 2024 Jul 16;18(7):e0012287. doi: 10.1371/journal.pntd.0012287 (PMC11251603; doi:10.1371/journal.pntd.0012287)
Supplement: Attachment — Submitted filename: Reviewer-PNTD response-2(1f).docx [file pntd.0012287.s002.docx]

**Response to Reviewer’s comments**

First, we would like to thank the editor and reviewers for spending their time in carefully going through the manuscript and providing scholarly comments that are believed to enrich the manuscript. Below are our point-by-point responses to the reviewer’s comments that are also incorporated in the main manuscript highlighted by green color.

**Reviewer #1:**

**Comment #1**: The title is captured as a national wide study yet sampling was only done in a few abattoirs and a pastoral area. A national wide study would mean that livestock population densities in the entire county was considered and weighted during sampling to capture regional differences, spatial distributions etc. Consider revising the title.

**Response to comment #1:**

We appreciate the reviewer professional comment, which is acceptable, and we revised the title as follows: A sero-epidemiological analysis of *Coxiella burnetii* infection and its risk factors in livestock from Addis Ababa, Adama, and Modjo abattoirs and pastoral areas of Oromia, Ethiopia

**Comment #2**: English editing is recommended for the manuscript.

**Response to comment #2:**

We accepted the reviewer comment and accordingly we did editing for the manuscript as much as possible.

**Comment #3:**

The authors should re-check the whole manuscript and remove redundant information in the abstract, author summary and many other sections.

**Response to comment #3:**

We thank the reviewer for his/her thorough checking of our manuscript. We checked the whole manuscript as per the recommendation of the reviewer.

**Comment #4:**

- Line 55 ‘‘prevalence rate’’ change to ‘prevalence estimate’

- Line 58-60: Please re-structure the sentence ‘‘The seroprevalence at abbatoirs and pastoral areas was found in the order of Addis Ababa (27.17%)>Adama (19.41%)>Modjo (19.13)>pastoral areas (12.1%), respectively’’.

**Response to comment #4:**

We appreciate the reviewer comment, which is acceptable, and we did amendment accordingly to the manuscript.

**Comment #5:**

- Line 101-112: The information presented here is unreferenced.

- Lines 118- 123. Consider revising this paragraph as there are a number of repeated texts e.g., sampled species and age of sampled animals.

- Line 124: Briefly, explain how the age of the study animals was estimated.

- Lines 117. Consider deleting the word design as this is repeated in line 133.

- Lines 122- 123. Consider moving the information about sample size to the section ‘sample size determination’ as this information is again repeated.

- Lines 127-128: Explain the selection criteria for the households. How were households selected?

**Response to comment #5:**

We thank the reviewer for his/her technical comments to improve the quality of our manuscript. We accepted all the comments and made the necessary correction and clarification as highlighted with green color in the main manuscript.

**Comment #6:**

- Line 139: It’s important to clarify that the expected prevalence of *C. burnetii* was assumed to be 50% for all targeted livestock species. Please also provide a justification for this.

- Line 144: The included total number of animals (n = 4140) is approximately 2.7 times the estimated required sample size (n = 1536). Is there a justification why for this?

**Response to comment #6:**

We appreciate the reviewer comment. As we explained under introduction section, there were few studies in the country; we assumed the expected prevalence of *C. burnetii* to be 50% for each targeted livestock species purposely to increase our sample size so that at the end of the day our result becomes closer to the reality in order to minimize the error rate. Had we have resources and time, it would have been good if we screened all animals in the study area

**Comment #7:**

- Line 149: The uppercase should not be in the middle of a subheading.
- Lines 155-160: Please remove repeated texts. Eg line ‘Each sample was given a code to identify the specific animal’ repeated in line 159-160. Pease do this in the entire document to avoid redundancy.

**Response to comment #7:**

We thank the reviewer for thorough checking our manuscript during giving his/her comment. We checked the entire document and corrected it accordingly to avoid redundancy.

**Comment #8:**

- Lines 160-162: Clearly explain how serum was prepared. Was there centrifugation on site or a dedicated field laboratory?

**Response to comment #8:**

We thank the reviewer for his/her comment. We explained it as follows:

“The next morning tubes containing samples were centrifuged at 2000× g for 10 min at room temperature by taking them to the nearby veterinary clinic and serums were separated from the blood ….”

**Comment #8:**

- Lines 156-157: This information should be presented at the end of this section. Please explain how this animal-level information was collected?

**Response to comment #8:**

Thanks. We moved it to the end of the suggested section and explained the raised issue as follows: “Information about the individual animal such as its area, sex, age, species, and tick infestation, was recorded during the sample collection process by using sample information collection sheet prepared for this purpose”

**Comment #9:**

- Line 169-170: Please specify on whether the ELISA kit used detected antibodies to phase 1 and/or 11 antigens of *C. burnetii*.

**Response to comment #9:**

We appreciate the reviewer comment. The ELISA kit used was to detect antibodies to phase I antigens of *C. burnetii*

**Comment #10:**

- Line 181-184: Information about optical densities is repeated. Please revise accordingly. The information ‘‘the color development is directly proportional to the amount of *C. burnetii*’’ is unnecessary and should be deleted.

- Lines 184-192: Please be concise and consider removing unnecessary details in this section.

- Line 207: Remove the word infection.

- Line 207: The paragraph starting with ‘these maps can provide ……..’ should be moved to the discussion or conclusion sections.

**Response to comment #10:**

We appreciate the reviewer comment. We did revisions and correction as per the reviewers suggestion.

**Comment #11:**

- Lines 215- 216: ‘…….. estimate the effects of risk factors on the prevalence of *C. burnetii* infection’’. Consider re-writing this sentence. Did you mean to determine the risk factors associated with *C. burnetii* seropositivity?

- Lines 216- 217 ‘‘Descriptive statistics such as chi-squared test was to analyse the effect of individual factors on the prevalence of *C. burnetii* infection’’. This sentence should be corrected. Chi-squared test is a non-parametric test used to determine the independent associations between categorical factors and a dependent variable.

- Lines 221-222: ‘‘The measure of association between prevalence and risk factors was reported as the odds ratio’’. This information is unnecessary and can be removed.

- Line 223: ‘A P value of < 0.05 and a 95% confidence interval were considered to be significant in this study’. Perhaps better indicate that variables with p values ≤ were considered statistically significant in the…...

- Lines 222-224: The use of multilevel mixed-effects generalized linear models is not appropriate to your data given that a cluster sampling design was not used in this study or its not mentioned in the manuscript. What variable was used as a random effect in the modeling? More details on how data were fitted to these mixed effect models were conducted is not provided in the paper. Again, the results from these analyses were not presented.

**Response to comment #11:**

We appreciate the reviewer for thorough checking our manuscript. Sorry for the wrong terminologies/sentences we used. We corrected them by appropriate words and removed the unnecessary sentences from the main document.

**Comment #12:**

- Lines 218: Were there interactions among the correlates in the multivariable model Please provide more details on the risk factor modelling methodology used. How did the presented multivariable model compare with a null intercept and parsimonious model without non-significant factors e.g., animal’s sex. How did you select the final model?

**Response to comment #12:**

We thank the reviewer the valuable comment. As per the comment the multivariable logistic regression model was built manually by inclusion of each variable one by one. The non-significant variable was removed from the model and checked for confounding by looking at the percentage change in the coefficient of variable (s) that remain in the model.

**Reviewer #2**

**Comment #1:**

- Page 10. Table 1: Please create a column and provide the results of chi-squared test, the preliminary test done before multivariable logistic regression analyses.

**Response to comment #1:**

We appreciate the reviewer for the valuable comment to improve our manuscript. We did as per the comment and highlighted by green color in the main document.

**Comment #2:**

- Lines 229: Could you estimate the true prevalence estimates based on the sensitivity and specificity of the commercial ELISA kit used?

**Response to comment #2:**

We appreciate the reviewer for his/her professional comment. Accordingly, we calculated the true prevalence of *C. brnetii* infection based on the sensitivity (99%) and specificity (98%) of the commercial ELISA kit we used using the formula previously described by Farrokh et al. [42] as follows: True prevalence = (Apparent prevalence + Sp - 1)/(Se + Sp - 1). Hence,

True prevalence = (18.77% + 98%-1)/(99%+98%-1) = (0.1877+0.98-1)/(0.99+0.98-1) = 0.1677/0.97 = 0.17288 = 17.29%.

Thus, we updated our manuscript with these information highlighted by green color in the main document.

**Comment #3:**

- Line 248: use lower case for ‘p’ standing for p values in text.

- Line 249: Remove the word ‘infection’. Lines 249-253: consider revising this section. For example, when you say … the odds of infection in camels was 3.22 (95% CI, 2.57-4.05%) times that of cattle….. You only detected antibodies to *C. burnetii* and no other diagnostic methods were used to determine if the animals had active infection. The presence of antibodies indicates prior exposures and not necessarily active infections. Please revise accordingly all the relevant sections.

- Line 413: Please reference the presented supplementary file in the manuscript.

- The author should avoid repeating prevalence estimates in conclusion section.

**Response to comment #3:**

We appreciate the reviewer for thorough checking our manuscript. We made the necessary edition, revision and correction as per the reviewers comment

**Comment #4:**

- Include a section on study limitation in the discussion section e.g., potential cross-reactions etc.

**Response to comment #4:**

We thank the reviewer for his/her valuable comments. We included the limitation our study in one paragraph under the discussion section as follows:

“Our study has some limitations. We used an indirect ELISA to test the presence of antibodies against *C. burnetti* and could not distinguish between historical exposure and active infections. Additionally the tests used had less than 100% sensitivity and specificity which could pose a risk of misclassification. Our study did not include environmental factors as covariates when investigating factors associated with Q-fever antibody seropositivity, which may have accounted for some of the observed variations across different study sites. Furthermore, serological cross-reactions might have occurred between *C*. *burnetii* and other bacteria”
